# Loss of functional Dicer in mouse radial glia cell-autonomously prolongs cortical neurogenesis^☆^

**DOI:** 10.1016/j.ydbio.2013.08.023

**Published:** 2013-10-15

**Authors:** Tomasz Jan Nowakowski, Karolina Sandra Mysiak, Timothy O‘Leary, Vassiliki Fotaki, Thomas Pratt, David Jonathan Price

**Affiliations:** Centre for Integrative Physiology, University of Edinburgh, Hugh Robson Building, George Square, Edinburgh EH8 9XD, UK

**Keywords:** Dicer, microRNA, Neurogenesis, Telencephalon, Mouse

## Abstract

Radial glia of the mouse cerebral cortex emerge from neuroepithelial stem cells around embryonic day 11 and produce excitatory cortical neurons until a few days before birth. The molecular mechanisms that regulate the end of cortical neurogenesis remain largely unknown. Here we investigated if the Dicer-dependent microRNA (miRNA) pathway is involved. By electroporating a cre-recombinase expression vector into the cortex of E13.5 embryos carrying a conditional allele of *Dicer1*, we induced mosaic recombination causing *Dicer1* deletion and reporter activation in a subset of radial glia. We analysed the long-term fates of their progeny. We found that mutant radial glia produced abnormally large numbers of Cux1-positive neurons, many of which populated the superficial cortical layers. Injections of the S-phase marker bromodeoxyuridine between postnatal days 3 and 14 showed that much of this population was generated postnatally. Our findings suggest a role for Dicer-dependent processes in limiting the timespan of cortical neurogenesis.

## Highlights

• Analysis of radial glia development in the absence of functional Dicer.
• Loss of Dicer prolongs mouse cortical neurogenesis postnatally.
• We found no defects in the onset of gliogenesis.

## Introduction

Radial glia are a class of undifferentiated cerebral cortical progenitor cells that give rise to excitatory neurons and astrocytes (Miyata et al., 2001, 2010; Noctor et al., 2001; Okano and Temple, 2009; Voigt, 1989). Cortical neurogenesis in the mouse normally occurs between E11 and E17, after which radial glia generate mainly astrocytes (Caviness, 1982; Gillies and Price, 1993; Levers et al., 2001; Takahashi et al., 1995). Prolongation of the neurogenic period is believed to have coincided with neocortical expansion in mammals (Kriegstein et al., 2006). It is likely that modifications to the molecular mechanisms controlling the timing of the switch from radial glial neurogenesis to gliogenesis were instrumental in neocortical evolution. While the switching mechanism is of great interest, it still remains relatively obscure (Miyata et al., 2010; Okano and Temple, 2009).

MicroRNAs (miRNAs) are short non-coding RNA molecules that fine-tune gene expression by inhibiting the translation of target mRNAs. Most primary miRNA gene transcripts (pri-miRNAs) are generated by RNA polymerase II. Hairpins containing mature miRNAs (pre-miRNAs) are released from pri-miRNAs by endoribonucleolytic cleavage by Drosha in the nucleus (Lee et al., 2003). Pre-miRNA hairpins are exported into the cytoplasm and cleaved by Dicer (Bernstein et al., 2003; Nicholson and Nicholson, 2002) into mature miRNA and loaded into the RNA-induced silencing complex (Hutvagner and Zamore, 2002; Mourelatos et al., 2002), where they interact with target sequences in the mRNAs and induce post-transcriptional gene silencing. While most studies identifying miRNA–mRNA target interactions in mammalian systems report only moderate effects of miRNA action on protein levels (Baek et al., 2008), genetic ablation of the miRNA biosynthesis pathway causes severe abnormalities early in embryonic development (Bernstein et al., 2003). Several studies investigated the roles of Dicer-dependent processes in cortical development using conditional genetic removal of the RNase IIIb domain of Dicer in the forebrain with Cre-recombinase driven from *Foxg1*, *Nes*, *Nex* and *Camk2* promoters (Davis et al., 2008; De Pietri Tonelli et al., 2008; Kawase-Koga et al., 2009, 2010; Li et al., 2011; Makeyev et al., 2007; Nowakowski et al., 2011) as well as the *Hes5* regulatory elements (Knuckles et al., 2012). In all of these *in vivo* studies, removal of functional Dicer was found to result in an onset of apoptotic cell death, defects in radial glia specification and survival, defects in differentiation, migration and maturation of cortical neurons. Reduced viability of Dicer-deficient cortical progenitors was recently proposed to be associated with the loss of trophic factor support (Andersson et al., 2010). Consistent with this finding, studies inducing Dicer deletion in a small proportion of radial glial or neuronal cells *in vivo* found that apoptotic cell death is not always an inevitable consequence of Dicer loss and that Dicer-deficient cells can survive when surrounded by wild-type cells (Cuellar et al., 2008; Nowakowski et al., 2013), offering the opportunity to examine other defects.

Here we extended our analysis of the cell autonomous functions of miRNAs in cortical progenitors. Lineage analysis revealed that radial glia lacking functional Dicer produced abnormally large numbers of *Dicer1*^*−/−*^ neurons in the postnatal cortex. Further investigation of this surprising finding revealed that many of these *Dicer*^*−/−*^ neurons were generated after the normal cortical neurogenesis period.

## Materials and methods

The licence authorising this work was approved by the University of Edinburgh Ethical Review Committee of 22nd September 2008 (application number PL35-08) and by the Home Office on 6th November 2008. Animal husbandry was in accordance with the UK Animals (Scientific Procedures) Act 1986 regulations. Mice homozygous for *Dicer1*^*fl*^ (Harfe et al., 2005) were crossed to mice carrying the *Rosa26R:YFP* (*R26RYFP*) transgene (Srinivas et al., 2001). Experimental embryos were generated and electroporated *in utero* as described before (Nowakowski et al., 2013; Saito, 2006). Bromodeoxyuridine (BrdU) (Sigma) was administered intraperitoneally (50 µg/g body weight). The cre-recombinase construct was kindly provided by Anjen Chenn, Northwestern University. Embryonic brains were fixed in 4% paraformaldehyde (PFA). Postnatal animals were perfused transcardially with 0.1 M phosphate buffered saline pH 7.4 (PBS) and 4% PFA for histology. Tissue was cryoprotected with 15% sucrose in PBS, equilibrated in 15% sucrose in PBS/OCT (Fisher) for 1 h with constant agitation before freezing. Cryosections were cut at 20 µm and stored at −20 °C.

### Immunohistochemistry

Immunohistochemistry followed standard protocols with heat induced antigen retrieval (AR) achieved by microwaving the slides in 10 mM sodium citrate, pH=6.0. Primary antibodies included in this study were against: BrdU (rat, 1:50, Abcam), Dcx (rabbit, 1:1000, Abcam), Cux1 (rabbit, 1:50, Santa Cruz), GFP/YFP (goat, 1:400, Abcam), NeuN (mouse, 1:500, Chemicon), Sox9 (rabbit, 1:1500, Millipore), and Tbr2 (rabbit, 1:100, Abcam). Binding was revealed using an appropriate fluophore-conjugated secondary AlexaFluor antibody raised in Donkey (1:400, Invitrogen). Nuclear counterstain DAPI (Vector) was applied in PBS at 1:10000. Slides were mounted in Vectashield hard-set (Vector) or Prolong gold (Invitrogen).

### Electrophysiology

Brains of P14 pups were dissected in ice-cold buffer containing 86 mM NaCl, 1.2 mM NaH_2_PO_4_, 25 mM KCl, 25 mM NaHCO_3_, 20 mM glucose, 75 mM sucrose, 0.5 mM CaCl_2_, 7 mM MgCl_2_ and 300 μm coronal slices were prepared using a Leica VT1200S vibratome. Slices recovered for 30 min at 35 °C in artificial cerebrospinal fluid (aCSF) containing 124 mM NaCl, 1.2 mM NaH_2_PO_4_, 25 mM KCL, 25 mM NaHCO_3_, 20 mM glucose, 2 mM CaCl_2_, and 1 mM MgCl_2_. For recording, slices were visualised using infra-red DIC/epifluorescence optics using an Olympus BX51WI upright microscope and continuously perfused with oxygenated aCSF containing picrotoxin (50 µM) at 33–35 °C. Whole-cell recordings were obtained using an Axon Multiclamp 700B amplifier and a custom National Instruments data acquisition/analysis system. Patch-pipettes (resistance 3–5 MOhm) were filled with an internal recording solution containing 130 mM K-methylsulphonate, 10 mM KCl, 10 mM HEPES, 0.1 mM EGTA, 10 mM glucose, 10 mM Na-phosphocreatine, 4 mM Mg-ATP, 0.5 mM Mg-GTP, and 5 mM Alexa-555 disodium salt, (pH 7.3 with KOH; 290–300 mOsm). Neuron-like YFP-positive cells in layer II/III of the cortex were visually identified and once patched their identity was confirmed by dialysis with Alexa-555. Series resistance was monitored throughout each experiment; only experiments where series resistance was <25 MOhm and varied <15% were included for analysis.

### Imaging and quantification

Immunofluorescent sections were imaged using either a Leica microscope connected to a Leica DFC 360 FX digital camera or a Zeiss LSM 150 confocal system. Image analysis including image intensity, area, cortical thickness or distance measurements were performed either in ImageJ or in MatlabR2009a (Mathworks). For embryonic sections, optical sections through the dorso-lateral telencephalon containing electroporated cells were acquired at constant separation. Counting ladders consisting of 200 µm (wide)×40 µm (deep) boxes were positioned in Adobe Photoshop with the base along the white matter. For postnatal sections, brains of all surviving mice were sectioned and analysed for GFP expression. Only brains with GFP+ cells in the dorso-lateral cortex were considered. Counting ladders consisting of 500 µm×100 µm boxes were positioned with the base along the pial edge. For quantification of Doublecortin (Dcx) positive cells, single 400 µm wide box spanning the cortical wall was used. Borders between cortical layers were established based on nuclear counterstaining with DAPI. Student's *t*-tests were performed using Microsoft Office Excel; Tukey's tests and three-way ANOVA were performed using Sigmastat (SYSTAT Software). In all figures: *—*p*<0.05, **—*p*<0.01, and ***—*p*<0.001.

## Results

### Dicer deficient progenitors generate more cortical neurons

**Fig. 1:**
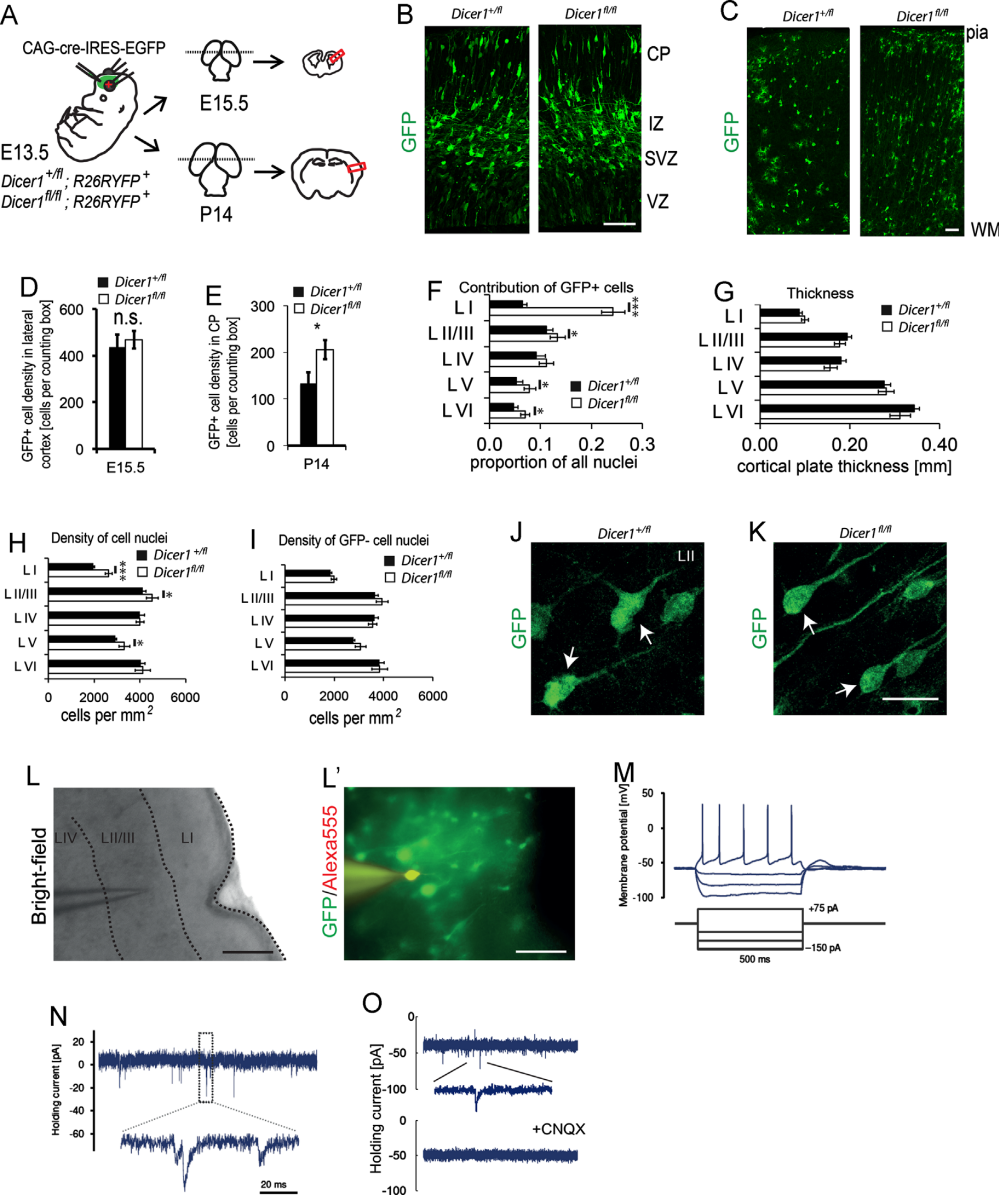
Loss of functional Dicer1 causes overproduction of cortical neurons by P14. (A) The experimental design: *Dicer1*^*fl/fl*^ and control *Dicer1*^*fl/+*^ embryos carrying the *R26RYFP* reporter allele were electroporated with cre-expression vector at E13.5 and developed until E15.5 or P14. Cells derived from electroporated radial glia were found reproducibly in the lateral cortex (red box). (B) At E15.5, they contributed to the ventricular zone (VZ), subventricular zone (SVZ), intermediate zone (IZ) and the cortical plate (CP), with high densities migrating in the IZ. (C) At P14, they contributed throughout the cortical wall (scale bars in C and D are 50 µm). (D) At E15.5, average densities of GFP+ cells in the developing cortex did not differ between genotypes (*p*=0.64, student's *t*-test, *n*=8 animals, from three surgeries, 2 sections counted per brain). (E) At P14, average densities of GFP+ cells were significantly higher (*p*=0.041, student‘s *t*-test, *n*=6 animals, 5 sections counted per brain) in *Dicer1*^*fl/fl*^ cortices. Data in D represent means±s.e.m. *n*=9 animals from 3 surgeries with 1 section analysed per animal. Data in E represent means±s.e.m. *n*=6 animals from 3 surgeries with 5 sections analysed per animal. (F) At P14 the contribution of GFP+ cells to cortical layers I, II/III, V and VI was higher in *Dicer1*^*fl/fl*^ than in *Dicer1*^*fl/+*^ mice. (G) Average thicknesses of cortical layers were not different between genotypes. (H) Average densities of cell nuclei were higher in layers I, II/III and V of cortices containing *Dicer1*^*fl/fl*^ cells. (I) Average densities of GFP− cells were not different between genotypes. Data in F–I represent means±s.e.m. *n*=6 animals from 3 surgeries with 5 sections analysed per animal; borders between cortical layers were identified with DAPI nuclear counterstain. (J) Cells generated from *Dicer1*^*+/−*^ and (K) *Dicer1*^*−/−*^ radial glia were able to adopt neuronal morphologies (arrows). Scale bar, 25 µm. (L–O) To validate that *Dicer1*^*−/−*^ GFP+ cells can develop into functional neurons, whole-cell patch clamp recordings were obtained from layer II/III GFP+ cells at P14 (*n*=6 cells from 4 animals from separate surgeries). (L) Wide-field image showing the position of recording electrode. (L′) A layer II/III GFP/YFP-expressing *Dicer1*^*−/−*^ cell filled with AlexaFluor dye following the recording. Scale bar, 100 µm. (M) Depolarisation of a patched layer II/III *Dicer1*^*fl/fl*^ neuron causes the neuron to fire action potentials. (N) Whole cell patch-clamp recording from a representative layer II/III *Dicer1*^*fl/fl*^ neuron receiving synaptic input. (O) Postsynaptic currents were sensitive to CNQX (5 μM, *n*=3 cells examined).

We tested the effects on postnatal day 14 (P14) lateral cortex of electroporating cre-recombinase expression vector (CAG-cre-IRES-EGFP) into E13.5 *Dicer1*^*fl/fl*^; *Rosa26RYFP*^*+*^ or *Dicer1*^*+/fl*^; and *Rosa26RYFP*^*+*^ (control) embryos (Fig. 1A–C). The progeny of Dicer-deficient radial glial progenitors were identified by immunohistochemistry using an antibody against both EGFP and YFP (Fig. 1B and C, immunopositive cells will be referred to as GFP+ hereafter, see Materials and Methods). GFP+ cells isolated two days after electroporation by fluorescent activated cell sorting were shown by PCR to carry the deleted *Dicer1* allele with no evidence of residual wild-type allele. We demonstrated previously that this deletion results in a rapid depletion (within 24 h) of *Dicer1* mRNA and mature miRNAs in electroporated cells (see Supplemental Fig. 1 in Nowakowski et al., 2013). Two days after E13.5 electroporation, we found no statistically significant difference in the average densities of GFP+ cells in the cortex of control and *Dicer1*^*fl/fl*^ embryos (Fig. 1D), suggesting that transfection efficiencies following electroporation were consistent between embryos of the two genotypes. By P14, control and *Dicer1*^*fl/fl*^ mice contained GFP+ cells throughout the cortical depth (Fig. 1C) with systematically higher densities of GFP+ cells in *Dicer1*^*fl/fl*^ mice than in controls (Fig. 1E). Greater proportions of GFP+ cells were found in *Dicer1*^*fl/fl*^ mice, particularly in layer I, but also in layers II/III, V and VI, than in controls (Fig. 1F). This increased contribution of *Dicer1*^*−/−*^ GFP+ cells in *Dicer1*^*fl/fl*^ mice did not increase the average thickness of the cortical layers (Fig. 1G) but did increase the average density of cells in most layers, most notably in the superficial layers I and II/III but also in layer V (Fig. 1H). The effects were limited to the mutated cells: there were no differences in the average densities of GFP− cells between *Dicer1*^*fl/fl*^ and control mice (Fig. 1I).

We examined the nature of the abnormally large population of *Dicer1*^*−/−*^ GFP+ cortical cells generated particularly in superficial layers. Many *Dicer1*^*−/−*^ GFP+ cells had neuron-like morphologies (Fig. 1J, and K). P14 *Dicer1*^*−/−*^ cells fired fast, regenerating action potentials upon depolarisation (Fig. 1L, L′, and M), had passive electrical properties normal for fast-spiking excitatory cortical neurons (Fig. 1N) and exhibited spontaneous excitatory postsynaptic currents that were sensitive to CNQX (Fig. 1O). These observations indicate that *Dicer1*^*−/−*^ cells can differentiate to form functional neurons capable of responding to synaptic input.

### *Dicer1*^*−/−*^ cortical progenitors continue to generate increased numbers of cortical neurons postnatally

**Fig. 2:**
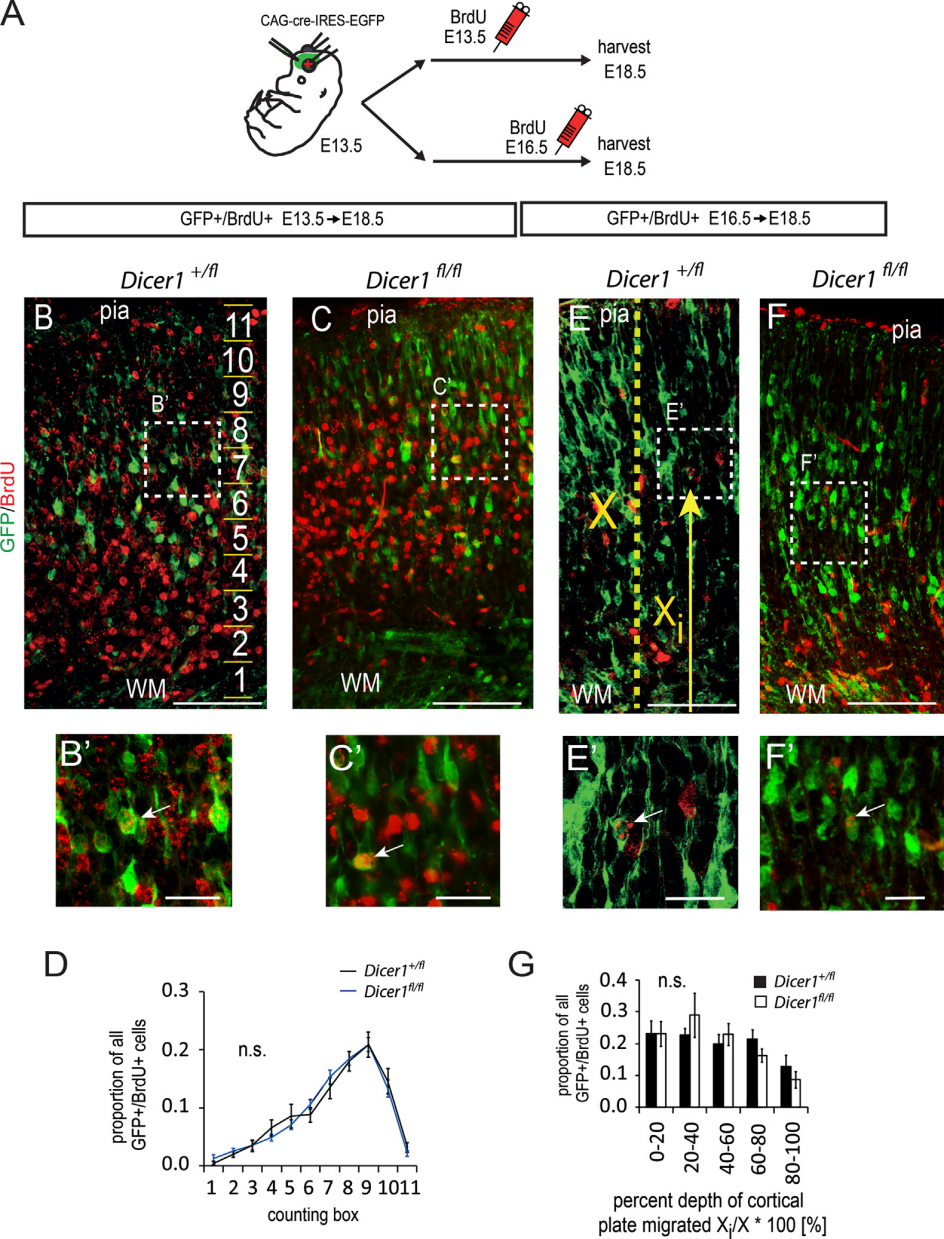
Loss of mature miRNAs does not affect the migration of embryonically-generated postmitotic neurons. (A) Experimental design: E13.5 *Dicer1*^*fl/fl*^*;R26RYFP*^*+*^ or *Dicer1*^*fl/+*^*;R26RYFP*^*+*^ embryos were electroporated with cre-expression vector and exposed to a pulse of BrdU on E13.5 or E16.5 and sacrificed on E18.5. (B and C) Representative images of coronal sections through the cortical plate of (B) *Dicer1*^*fl/+*^ and (C) *Dicer1*^*fl/fl*^ embryos pulse-labelled with BrdU on E13.5. A counting ladder composed of 11 counting boxes was used to quantify the relative distribution of GFP+/BrdU+ cells (C: 50 µm between steps). Scale bar, 100 µm. (B′ and C′) Higher magnification images showing examples of GFP+ cells which retained BrdU (arrows) from lower magnification images in B and C. Scale bar 25 µm. (D) Relative distribution at E18.5 of GFP+ cells that were labelled with BrdU given at E13.5 shows no difference between genotypes (data represent means±s.e.m., *p*-value calculated using Tukey's test for 10 control and 12 experimental embryos from 3 surgeries, 5 sections analysed per brain). (E and F) Images of E18.5 cortical plates from (E) *Dicer1*^*fl/+*^ and (F) *Dicer1*^*fl/fl*^ embryos immunolabelled for GFP and BrdU after a pulse given at E16.5. Scale bar 50 µm. (E′, F′) Higher power magnification images showing GFP+ cells which retained BrdU (arrows). Scale bar 25 µm. (G) Per cent depth of cortical plate migrated was calculated by measuring the distance ‘*X*_i_’ of each cell from the white matter (WM) as indicated by arrow in E and expressed as a per cent of the thickness of the cortical plate ‘*X*’ as shown by the broken line in E. There was no difference in the distribution of GFP+/BrdU+ double labelled cells between genotypes (data represent means±s.e.m., *n*=7 control and 5 experimental embryos from 3 surgeries, 8 sections analysed per brain).

Comparison of the embryonic migration of *Dicer1*^*−/−*^ and control postmitotic neurons using pulses of bromodeoxyuridine (BrdU) at E13.5 or E16.5 (Fig. 2A) revealed no differences in their distributions by E18.5 (Fig. 2B–G). This result suggested that the explanation for the large postnatal increase in *Dicer1*^*−/−*^ neurons might be found after birth. We tested whether increased neurogenesis persists postnatally among *Dicer1*^*−/−*^ cortical cells.

**Fig. 3:**
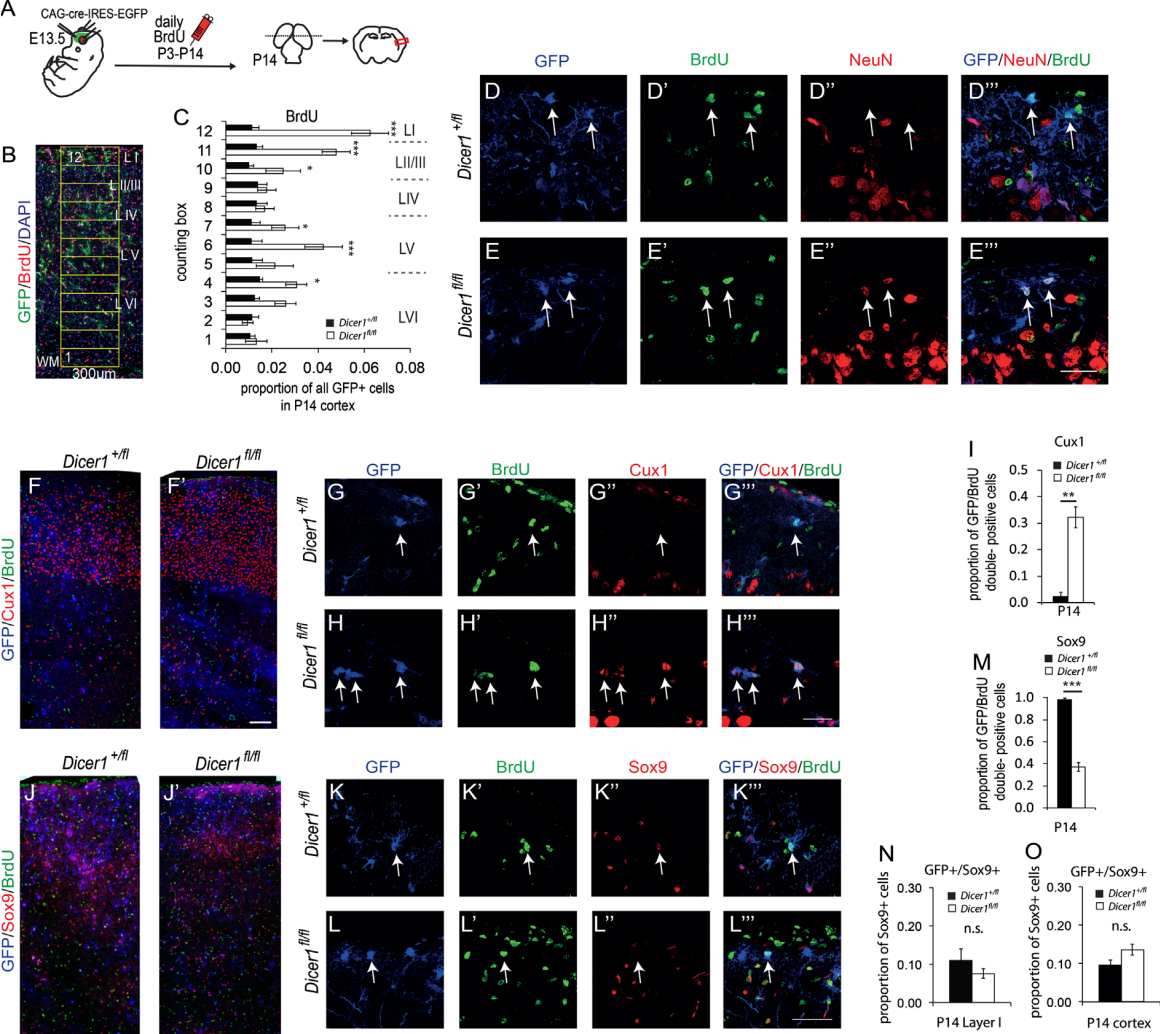
Increased postnatal neurogenesis following the loss of Dicer. (A) After cre-expression vector electroporation at E13.5, *Dicer1*^*fl/fl*^*;R26RYFP*^*+*^ and *Dicer1*^*fl/+*^*;R26RYFP*^*+*^ pups aged P3–P14 received daily injections of BrdU. (B) Distribution of GFP+/BrdU+ cells was quantified using a counting ladder (twelve 300 µm × 100 µm boxes). (C) Significantly higher proportions of GFP+ BrdU+ cells were found in layers I, II/III, V and VI in *Dicer1*^*fl/fl*^ animals than in controls (data represent means±s.e.m, *n*=6 animals from 3 surgeries of each genotype, 2 sections counted per animal). (D–E″′) Representative images showing layer I of electroporated *Dicer1*^*fl/+*^ and *Dicer1*^*fl/fl*^ embryos immunolabelled for GFP, BrdU and NeuN: (D″′) in controls, GFP+ BrdU+ cells were NeuN-negative (arrows); (E″′) in *Dicer1*^*fl/fl*^ mice, many triple-labelled cells were found (arrows). (F and F′) Electroporated *Dicer1*^*fl/+*^ and *Dicer1*^*fl/fl*^ cortices were immunolabelled for GFP, BrdU and Cux1 to identify upper cortical layer neurons born postnatally. (G–H″′) High magnification images showing layer I of electroporated *Dicer1*^*fl/+*^ and *Dicer1*^*fl/fl*^ cortices immunolabelled for GFP, BrdU and Cux1: (G″′) in controls, most GFP+ BrdU+ cells were Cux1-negative (arrow); (H″′) in *Dicer1*^*fl/fl*^ embryos, many triple-labelled cells were found. (I) Quantification shows that many more postnatally born *Dicer1*^*−/−*^ than control cells expressed Cux1. (J and J′) Electroporated *Dicer1*^*fl/+*^ and *Dicer1*^*fl/fl*^ cortices were imunolabelled for GFP, BrdU and Sox9 to identify the glial population born postnatally. (K–L″′) High magnification images showing layer I of electroporated *Dicer1*^*fl/+*^ and *Dicer1*^*fl/fl*^ embryos immunolabelled for GFP, BrdU and Sox9: (K″′) a triple- labelled cell in a control (arrow); (L″′) in *Dicer1*^*fl/fl*^ embryos, many GFP+ BrdU+ cells were not immunoreactive for Sox9 (arrow). (M) Quantification shows fewer postnatally born *Dicer1*^*−/−*^ than control cells expressed Sox9. Scale bars in E″′, G″′, J″′ 25 µm. Data in I, M represent means±s.e.m., *n*=3 animals, three sections per animal. (N,O) Graph showing the proportion of GFP and Sox9 double-positive cells expressed as a fraction of all Sox9 positive cells in (N) layer I or (O) all cortical layers. Data represent means±s.e.m., *n*=6 animals.

We administered a daily pulse of BrdU to electroporated *Dicer1*^*fl/fl*^ and control *Dicer1*^*fl/+*^ animals between P3 and P14 (Fig. 3A). Proportions of GFP+ cells that were BrdU+ were higher in *Dicer1*^*fl/fl*^ cortices than in control cortices, with significant increases of up to six-fold particularly in layer I and also in layers II/III, V and VI (Fig. 3B and C), corresponding with the increased contribution of *Dicer1*^*−/−*^ GFP+ cells to these layers shown in Fig. 1F. The average densities of GFP− BrdU+ cells were not different between genotypes (not shown). In *Dicer1*^*fl/fl*^ cortices (*n*=3), many BrdU+ GFP+ double-positive cells were positive for the neuronal marker NeuN (Fig. 3E–E″′, arrows), whereas we never observed such triple-labelled cells in control cortices (*n*=3) (Fig. 3D–D″′). To distinguish astroglial and neuronal populations, cells were immunolabelled for neuronal marker Cux1 and astrocyte marker Sox9. In controls, almost all BrdU+ GFP+ cells in layer I expressed Sox9 but very few expressed Cux1 (Fig. 3F, G–G″′, I, J, K–K″′, and M), whereas about a third of *Dicer1*^*−/−*^ BrdU+ GFP+ cells in layer I expressed Cux1 and about 40% expressed Sox9 (Fig. 3 F′, H–H″′, I, J′, L–L″′, and M). It is likely that the reduction in the proportions of *Dicer1*^*−/−*^ GFP/BrdU double-positive cells that expressed Sox9 (Fig. 3M) occurred because a greater proportion of these double-positive cells were neuronal, rather than there being an absolute reduction in the generation of astroglia from *Dicer*^*−/−*^ cells. To test this, we compared the average fractions of the Sox9+ cell populations that expressed GFP in the electroporated regions of *Dicer1*^*fl/fl*^ and *Dicer1*^*+/fl*^ cortices. The vast majority, about 90%, of Sox9 cells were non-electroporated (i.e. GFP−) and there was no evidence that their densities were affected by intermingled electroporated (GFP+) cells (Fig. 3J, J′, N, and O). The average contributions (about 10%) of *Dicer1*^*fl/fl*^ and *Dicer1*^*+/fl*^ GFP+ cells to the glial populations did not differ significantly between genotypes, neither specifically in layer I (Fig. 3N), which has the highest BrdU+ GFP+ cell contribution, nor overall in the cortex (Fig. 3O). These results suggest that prolonged cortical neurogenesis did not occur at the expense of gliogenesis.

**Fig. 4:**
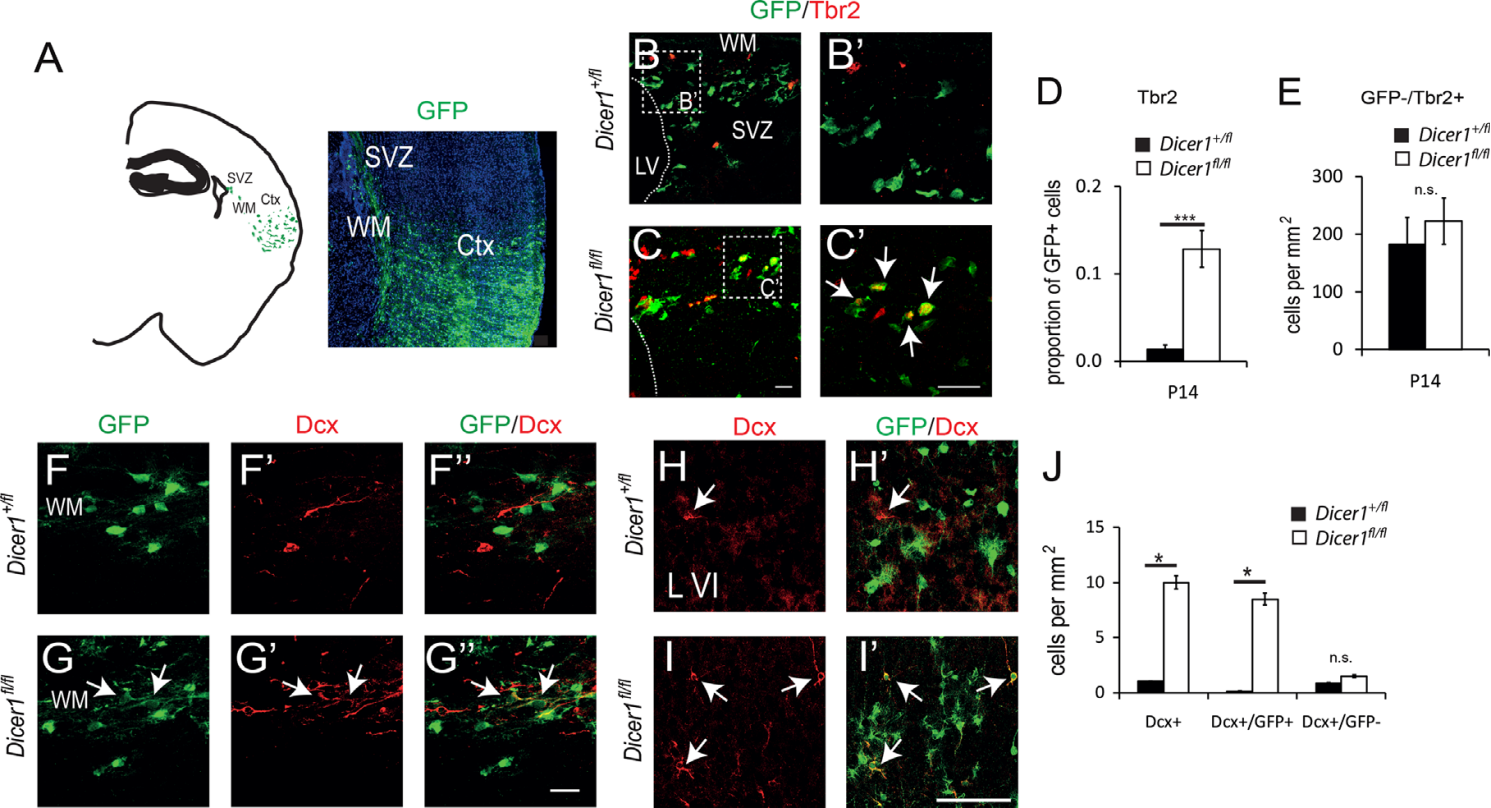
Increased numbers of Tbr2-expressing cortical cells and migrating neuroblasts persist until P14 following the loss of functional Dicer. (A) The distribution of GFP+ cells typically seen in the cortex (Ctx), along the white matter (WM) and in the subventricular zone (SVZ) of both *Dicer1*^*fl/fl*^*;R26RYFP*^*+*^ and *Dicer1*^*fl/+*^*;R26RYFP*^*+*^ animals at P14. (B and B′) In *Dicer1*^*fl/+*^ SVZ at P14 most GFP+ cells do not express Tbr2. (C and C′) In *Dicer1*^*fl/fl*^ SVZ, GFP+ and Tbr2+ double-labelled cells are easily detectable (arrows in C′). Scale bars B–C′: 50 µm. (D) A higher proportion of GFP+ *Dicer1*^*−/−*^ than *Dicer1*^*+/−*^ cells expressed Tbr2 while (E) the density of GFP-/Tbr2+ cells in the SVZ was not different between genotypes. Data represent means±s.e.m; *n*=8 animals from 4 surgeries, 7–8 sections counted per brain. (F–G″) WM underneath the electroporated areas of P14 cortex stained for GFP and migrating neuroblast marker Doublecortin (Dcx). Control *Dicer1*^*+/−*^ GFP+ cells (F) show no staining for Dcx (F′ and F″). A number of *Dicer1*^*−/−*^ GFP+ cells (G) expressed Dcx (G′ and G″). Scale bar 25 µm. (H, H′) Most Dcx positive cells (e.g. arrow) in the cortex at P14 did not express GFP in control animals. (I, I′) Numerous Dcx+ cells (arrows) in *Dicer1*^*fl/fl*^ animals were double-positive for GFP, primarily in deep layers of the cortex. Scale bar 50 µm. (J) More Dcx expressing *Dicer1*^*−/−*^ than *Dicer1*^*+/−*^ cells were found per unit area of the electroporated cortex, while densities of Dcx+/GFP− cells were not different. Data represent means±s.e.m., *n*=6 animals from 3 surgeries.

We then examined the GFP+ cells in P14 brains, which were found in the SVZ, along the white matter (WM) and in the cortex (Ctx) in both genotypes (Fig. 4A), for persistent co-expression of makers of progenitors and immature neurons. We tested for expression of Tbr2, a marker of intermediate progenitors in the subventricular zone (SVZ) that generate most superficial layer neurons, and Doublecortin (Dcx), a marker of migrating neuroblasts. During normal postnatal cortical development, the Tbr2-expressing population decreases in size until only a few cells can be found in the SVZ by P14 (Fig. 4B, B′, and D). At P14, we found a greater proportion of GFP+ cells in the SVZ expressed Tbr2 in *Dicer1*^*fl/fl*^ animals than in control animals (Fig. 4C, C′, and D).

The persistence of an abnormally large population of Tbr2-expressing cells was accompanied by the presence of abnormal numbers of immature migrating neuroblasts, both in the white and the grey matter. In white matter underneath the electroporated region in control brains, we could not find *Dicer1*^*+/−*^ GFP+ cells expressing Dcx (Fig. 4F–F″′), whereas *Dicer1*^*−/−*^ GFP+ cells expressing Dcx were readily detectable (Fig. 4G–G″′). When we examined the overlying grey matter of controls at P14, we found very few cells expressing Dcx most of which were GFP− (Fig. 4H, H′, and J). Significantly more Dcx+ cells were found in *Dicer1*^*fl/fl*^ cortex and most of these Dcx+ cells co-expressed GFP (Fig. 4I, I′, and J).

In both cases the requirement for miRNAs was cell autonomous as there was no difference in the densities of surrounding Tbr2+/GFP− cells (Fig. 4E) or Dcx+/GFP− cells (Fig. 4J) in the cortex.

These data indicate that loss of Dicer significantly enhanced the production of neurons postnatally. They suggest that miRNAs normally cell-autonomously restrict the production and persistence of Tbr2-expressing intermediate cortical progenitor cells and hence limit the neuronal output of the cerebral cortex.

## Discussion

In this study we analysed a model of mosaic deletion of *Dicer* in a subset of radial glia and found an abnormally large population of cortical neurons generated outside the normal time-window of cortical neurogenesis. Furthermore, we observed abnormal maintenance of Tbr2-expressing intermediate progenitors in the SVZ of P14 cortex. Since intermediate progenitors are believed to produce neurons directly (Kowalczyk et al., 2009), it is likely that postnatal persistence of abnormally large numbers of intermediate progenitors accounts for the extended time-span of mouse cortical neurogenesis. Interestingly, a recent study reported increased numbers of proliferating and apoptotic cells in the dentate gyrus, and reduced numbers of mature hippocampal neurons (Li et al., 2011). This phenotype suggested that long-term loss of functional Dicer may extend the maintenance of undifferentiated neuronal progenitors, or prevent their entry into quiescence, in the hippocampal dentate gyrus, one of the regions where neuronal progenitors are maintained throughout life. Cortical neurogenesis takes place between E11 and E18 and to the best of our knowledge, no manipulation prolonging the maintenance of intermediate cortical progenitors or cortical neurogenesis beyond that period has so far been reported.

While canonical Drosha-Dgcr8-dependent pre-miRNAs remain the best studied substrates for Dicer, recent reports demonstrated that Dicer is also responsible for the cleavage of retrotransposon transcripts (Kaneko et al., 2011) as well as for the maturation of numerous non-canonical miRNAs, endogenous small interfering RNAs and short-hairpin RNAs (Babiarz and Blelloch, 2008; Babiarz et al., 2008; Castellano and Stebbing, 2013). Conditional removal of Dgcr8 from the developing cortex results in a less severe range of phenotypes compared to the loss of Dicer (Babiarz et al., 2011). It remains to be investigated whether loss of particular Dicer-dependent but Dgcr8-independent short RNAs contributes to Dicer knockout-specific phenotypes in the developing cortex.

An intriguing feature of our results was that many of the overproduced *Dicer*^*−/−*^ neurons were found in layer I by P14. This could be explained by the loss of miRNAs having a cell autonomous effect, for example by affecting the transcript levels of genes that promote neuronal migration or inhibit terminal differentiation. In line with this hypothesis, miR-134 was recently proposed to regulate migration and differentiation of cortical neurons (Gaughwin et al., 2011). A further contributing factor could be that Cajal–Retzius neurons, which safeguard layer I from invasion by new neurons during normal period of cortical neurogenesis (Hashimoto-Torii et al., 2008), disappear early in postnatal development (Chowdhury et al., 2010) and may therefore not be able to prevent the migration into layer I of *Dicer1*^*−/−*^ neurons born postnatally. Further studies will be needed to investigate the impact of cell autonomous loss of Dicer on terminal differentiation of cortical neurons by allowing some of the animals to develop to adulthood and looking at neurite outgrowth complexity and spine development. Several miRNAs have been implicated in these processes, including miR-124, miR-34a and miR-132 (Agostini et al., 2011; Clovis et al., 2012; Franke et al., 2012; Vo et al., 2005; Yu et al., 2008) and it will be interesting to see if loss of Dicer partially or completely recapitulates the defects seen following the specific loss of these miRNAs.

For two aspects of development, the formation of functional neuronal properties and *embryonic* migration, we did not detect abnormalities among the progeny of the electroporated *Dicer1*^*fl/fl*^ radial glia. The latter result contrasts with previous findings from studies using cre-recombinase expressing mice, which reported severe defects in neuronal migration (De Pietri Tonelli et al., 2008; Kawase-Koga et al., 2009; Nowakowski et al., 2011). A possible explanation is that in our mosaics the presence of numerous non-electroporated radial glial fibres may provide sufficient support to rescue many neuronal migration defects observed in models with more widespread deletion throughout the radial glial population. Another possibility is that residual Dicer1 and/or miRNAs persisting after deletion in our model might be sufficient for neuronal migration to occur over the following days. This would be hard to exclude.

Many molecular mechanisms have been implicated in the regulation of the switch from neuro- to glio-genesis of the radial glia, in particular the Notch and BMP signalling pathways and epigenetic mechanisms (Miyata et al., 2010). Various miRNAs have been shown to directly regulate the expression of genes involved in most of these diverse pathways (Fineberg et al., 2012; Gaughwin et al., 2011). While it is likely that the effects we describe here are caused by a loss of many miRNAs deregulating multiple pathways, loss of miRNAs regulating cell autonomous mechanisms are likely to be particularly important. Interestingly, it was suggested recently that miRNAs control the developmental ‘clock’ of radial glia neurogenesis, since early cortical ablation of *Dicer1* induced using *Emx1*^*cre*^ results in a sustained generation of deep instead of superficial cortical layer neurons (Saurat et al., 2013). This study also showed that early loss of functional Dicer in radial glia did not abolish or delay gliogenesis (Saurat et al., 2013), which is in agreement with our findings that Dicer-deficient radial glia contribute normally to the glial population by P14.

It is a strong possibility that at least some of the postnatally-born *Dicer*^*−/−*^ neurons are generated from *Dicer*^*−/−*^ Tbr2+ intermediate progenitors that are present postnatally in abnormally large numbers. This is suggested by our observation of increased numbers of Tbr2+ *Dicer*^*−/−*^ cells postnatally. In a previous study, we observed that mosaic loss of Dicer caused an increased production of Tbr2+ cells *prenatally* (Nowakowski et al., 2013). It is possible that an expanded population of *Dicer*^*−/−*^ Tbr2+ cells present at birth persists postnatally because its cells continue to self-renew rather than undergoing divisions that generate only postmitotic progeny. It is also possible that these cells persist because their cell cycle times become very long, or a combination of mechanisms may be responsible. It is even conceivable that, following the loss of *Dicer*, Tbr2 becomes re-expressed in either a quiescent cortical progenitor population or in postmitotic cells leading to their re-entry into the cell cycle. Further studies will be needed to investigate these interesting possible mechanisms. Recent studies implicated miR-92a/b in the regulation of intermediate progenitor cell specification in the mouse SVZ by regulating the expression of Tbr2 transcription factor (Bian et al., 2013; Nowakowski et al., 2013). It is possible that over-expression of Tbr2 due to the loss of miR-92a/b could to some extent account for the persistence of abnormally large numbers of progenitors that we observed postnatally. To the best of our knowledge, a study of the long-term postnatal effects of earlier Tbr2 gain-of-function has not been performed, but such a study would help to determine whether an early increase in the production of intermediate progenitor cells would extend the time-span of mouse cortical neurogenesis postnatally due to delayed postnatal depletion of that population.

Based on studies analysing periods of cortical neurogenesis in mouse, ferret and macaque it has been proposed that expansion of the SVZ and prolongation of cortical neurogenesis contributed to the evolutionary expansion of the neocortex (Kriegstein et al., 2006). In agreement with this hypothesis, time windows of cortical neurogenesis in ferret and macaque are, respectively, threefold and eight-fold longer than in mouse (Kornack and Rakic, 1998; Noctor et al., 1997), allowing for additional neurogenic divisions of cortical progenitors. Recent analysis of miRNA expression in developing human, chimp and macaque brains showed rapid changes in developmental expression profiles of a number of conserved miRNAs, including miR-92, in the primate lineage and proposed that miRNAs contributed to the brain's evolutionary expansion (Somel et al., 2011). It remains to be investigated what developmental processes might have changed due to altered miRNA expression in the developing primate brain. Our current analysis of the genetic ablation of miRNA production in the mouse radial glia demonstrates that miRNAs regulate the timespan of cortical neurogenesis and suggest a mechanism by which changes in miRNA maturation and/or in miRNA expression levels could contribute to the evolution of the cerebral cortex.
